# The Effects of a Dance and Music-Based Intervention on Parkinson’s Patients’ Well-Being: An Interview Study

**DOI:** 10.3390/ijerph19127519

**Published:** 2022-06-20

**Authors:** Barbara Colombo, Alison Rigby, Martina Gnerre, Federica Biassoni

**Affiliations:** 1 Behavioral Neuroscience Lab, Champlain College, Burlington, VT 05401, USA; al.rigby9@gmail.com; 2 Università Cattolica del Sacro Cuore, 20123 Milano, Italy; martina.gnerre@unicatt.it (M.G.); federica.biassoni@unicatt.it (F.B.)

**Keywords:** Parkinson’s, dance, music, well-being, interview, text analysis, content analysis

## Abstract

Previous research has shown the positive effects of music and dance-based interventions on the physical and psychosocial symptoms of Parkinson’s disease (PD). The aims of this study were: (1) to investigate how PD patients subjectively perceive the emotional, cognitive, and social benefits of a music- and dance-based intervention; (2) to apply an innovative methodology for an interview analysis combining findings from a linguistic text with an analytic approach and conducted with the software LIWC and from the content analysis performed by human coders. Extensive, open-ended interviews were conducted with 13 patients with PD who had participated in a dance and music program. The interviews were analyzed using both human coders and the computer-based approach. The results show that emotional and social aspects are considered the most frequent perceived benefits of the dance program. The data confirm the positive impact of dance- and music-based programs on promoting participants’ emotional and social well-being. A combined approach to text analysis appears to be a promising way to achieve more in-depth insights into patients’ subjective perceptions.

## 1. Introduction

Parkinson’s disease (PD) is a neurodegenerative disorder characterized by motor dysfunctions as its core clinical features. Its incidence is rising worldwide [1]: Accounting only for aging, approximately 700,000 PD cases have been predicted for 2040 in the United States alone [2]. When evaluating a larger sample from North America and adding different risk factors to the equation, the number increases to 1,238,000 by 2030 [3].

Considering this upward trend, and although recent genetic and pharmacotherapeutic advancements are promising, treatment providers are increasingly valuing and employing new nonpharmacological techniques and approaches to improve patients’ quality of life [4,5,6].

In a recent World Health Organization (WHO) review, Fancourt D. and Finn S. [7] highlight the importance of the performing arts (including therapeutic dance, dance therapy, and music therapy) in the prevention, management, and treatment of different illnesses [7]. In relation to dance, evidence-based research showed that both dance therapy and dance programs are effective interventions for health-related physical and psychological problems [8]. This seems particularly appropriate for Parkinson’s patients since if the main PD symptoms are linked to motor functions (e.g., rigidity, postural instability, rest tremor, balance difficulties, lack of coordination, and bradykinesia), numerous non-motor symptoms (such as cognitive dysfunction or mood/affective disorders) contribute to the progression of the overall disability and impaired quality of life, often to a greater extent than motor symptoms [9,10,11,12].

Dance interventions provide a pleasurable, cost-effective, and multidimensional experience where dancers have a chance to improve balance, coordination, visual-spatial ability, flexibility, imagery, creativity, rhythm, memorization, learning, social connection, and an overall enhancement in psycho-emotional well-being [8,9,13]. The positive effects of dance on the motor, cognitive and emotional dimensions in PD were demonstrated for the first time in a study published in 1989 [14]. Since then, we have seen an increase in dance-based intervention for PD patients. Specifically, when focusing on the mobility of individuals with PD, dance-based interventions have been reported to enhance walking speed [15], flexibility [16], pitch length, gait [17], balance [18], stability, coordination, body control [19,20], and to provide a better posture [17,21,22], as well as an overall heightened perceptual sensitivity [23]. The mobility improvement is both immediate (especially on limb rigidity, fine motor skills, and facial expression) [24] and is also reported as a long-term effect (especially when assessing improvements in balance, walking, activities of daily living, and physical fitness) [9,25,26,27].

Most of these studies seem to focus mainly on the physical symptoms of Parkinson’s, but dance-based interventions could be considered as one of the most promising approaches to address not only physical, but the cognitive and emotional symptoms associated with the progression of Parkinson’s disease [28]. For example, dance-based interventions targeting PD patients have been shown to improve motor learning [29], executive functions [30], memory [31], spatial cognition [30], and multitasking [32]. Few studies have focused directly on the emotional benefits of dance for PD patients, but we know from the literature that the combination of dance and music can reduce depression, anxiety, and stress levels, and may also enhance emotional understanding, empathy for others, emotional perception and expression, emotional interaction, and prosocial behaviors [33,34,35]. The combined effect of dance and music has been reported to be beneficial in addressing the physical and emotional symptoms of several neurodegenerative diseases [36,37,38], including PD [39], and these positive effects have been linked to the active participation required by these programs. Moreover, the fact that dance-based interventions include a music component adds the opportunity of improving social interaction, which has been reported to be enhanced by music-based social activities [40,41,42]. This is supported by the fact that dance programs have been shown to improve social interactions in clinical populations; patients involved in dance lessons are more engaged and motivated, and report perceiving a higher sense of community and belonging [43].

This cumulative set of studies provides support for the use of dance as an enriched and multidimensional environment to address the physical and psychosocial effects of PD, but little is known regarding the subjective perceptions of participants in the dance programs for PD.

Starting from these considerations, the first aim of this study was to investigate Parkinson’s patients’ perceptions of the benefits of a music and dance-based intervention. To be more specific, we were interested in exploring if patients would focus more on the specific motor benefits, if they would reflect or show emotional and cognitive benefits, and if they would reflect any appreciation of increased social interactions.

In order to achieve our aims, we first had to find the right program that would combine a focus on dance, music, and social interaction. A structured dance and music-based intervention that specifically targets PD patients is Movement for Parkinson’s, classified as a therapeutic dance program (i.e., a dance-based intervention intended to provide mental health or behavioral benefits in addition to physical/motor support, but that is not considered a form of psychotherapy) that aims at combining dance, imagery, and live music to address PD-specific issues, such as balance, flexibility, coordination, isolation, and depression. The structure (which highlights the importance of both the dance and music components) and the goals of the program, which intentionally focus on both physical and socio-emotional symptoms of PD, make this program the perfect ground to investigate participants’ beliefs and responses to this kind of intervention.

To be able to study participants’ beliefs and emotional and cognitive responses, we decided to rely on their free autobiographical narrations, as derived from open-ended interviews. The literature is consistent in highlighting that autobiographical narration is a preferred practice for re-elaboration and sense-making of the individual’s own experience, particularly for patients coping with medical diseases or interventions [44,45]. Nonetheless, to our knowledge, no research has been conducted using self-narratives about interventions aimed at empowering the well-being of patients affected by Parkinson’s disease. Within this frame, we relied on the assumption that language is the most common tool for converting internal thoughts and emotions into a form that others can understand. For this reason, psychologists and researchers use language as an ecological source of information to explore and understand individuals’ cognitive processes and feelings [46,47]. One methodological challenge for these qualitative studies is analyzing a large corpus of data in an accurate and reliable way. Traditionally, both technology-supported and manual approaches are used. However, it has also been suggested that combining these two methods can enhance the trustworthiness of the results [48].

Following this line of reasoning, the second aim of the study was to adopt this more in-depth analysis of the interviews to integrate the information that could be derived from a text analysis-based approach to the data derived from a content analysis (CA). For the text analysis, we used the LIWC software [49]; for the content analysis, we created an ad hoc coding scheme based on different quantitative text analysis approaches in psychology [50,51,52] and merged various content categories systems to get a specific coding system focused on the object of our investigation [53].

For our first aim, we were expecting to be able to highlight a strong focus on positive emotions, a mindful disposition derived from a focus on the present, and several links to social components within the patients’ interviews. For our second aim, we were expecting to be able to derive comparable information from the two approaches, although focusing on different aspects, and hence, be able to dive more in-depth into the interviews’ contents by combining the two approaches.

## 2. Materials and Methods

Ethical approval for this study was granted by Champlain College IRB (COA IRB000184).

### 2.1. Sample

We conducted 13 interviews. The interviews lasted between 742.20 and 4686 s [between 12 and 78 min] (Mean = 2234.40; SD = 1183.85; IQR = 1163.10–3024.90).

Our sample was balanced by gender (54% = females). Age was between 56 and 75 (Mean = 65.62; SD = 6.81; IQR = 60–73). All participants were English native speakers and were recruited during the class by presenting the research and asking for volunteers.

The participants who joined the study had a diagnosis of Parkinson’s from the UVM Hospital Center for Movement Disorders—the diagnosis was based on the medical team’s standardized protocols.

All participants who joined the study had been attending the classes regularly for at least 6 months at the time of the interview. The Movement for Parkinson’s program is held at a local performing arts center, although classes were transitioned to Zoom at the start of the pandemic. Classes (75 min in duration) are offered twice weekly by a certified Dance for PD instructor and caregivers are encouraged to attend. Dance for PD recognizes advanced skills, experience, and training through a special certification program for qualified dance teachers who have trained with them, have professional experience, and who most closely follow the class structure and artistic content of the Mark Morris Dance Group/Brooklyn Parkinson Group Dance for PD^®^ model.

Our sample size was deemed as adequate to help us reach information comprehensiveness and the consequent information power, a commonly used pragmatic guiding principle in qualitative research; it suggests that the more information power the sample provides, the smaller the sample size needs to be, and vice versa [54]. In our interviews, we were able to collect a large amount of data for each person, and our interviews were rich and experiential. As a consequence of these characteristics, fewer participants were needed to achieve high information power [55]. Finally, our study was characterized by strong and clear communication between researcher and participants, and this is another recognized feature that allows for fewer participants to provide enough information compared to a study with ambiguous or unfocused dialogue [54].

### 2.2. Procedure

Participants, after signing a consent form, were asked to tell the researchers, using their own words, about their experience with Movement for Parkinson’s. The interviews were audio-recorded and transcribed. The participants were also reminded that they did not have to disclose any personal detail or any identifying information and that any identifying information (names, specific locations, etc.) would be deleted from the transcripts. They were also told that they could ask for any section of their interview to be deleted prior to analysis (none of the participants asked for this).

### 2.3. Data Analysis–Content Analysis

In the first step of our analysis, we performed computerized content analysis using the Linguistic Inquiry and Word Count—LIWC 2015 software [49]. LIWC analyzes the content of a text in a sequential way. The software is programmed to match each word with the words of the reference dictionary that the researcher is using. For each match that the software identifies, the score of the corresponding category (or categories) is incremented. The values that the software returns represent the mean percentages of the words of the examined text for each target category. For example, a mean score of 5.32 for affective processes means that 5.32% of the words used by that individual were linked to affective processes (see Table 1 for examples).

LIWC software has been used by researchers in different areas of psychology in more than 100 studies [47,56]. These studies showed that LIWC is a reliable tool for text analysis focusing on emotional- and health-related processes; for example, it was able to identify (with scores similar to human coders) emotional expression that could predict subsequent patients’ visits to health centers [57] and COVID-related emotional outcomes [58].

In this study, we used the LIWIC 2015 standard dictionary, which includes almost 6400 words and stems, focusing on the categories reported in Table 1. A total of 76,291 words were analyzed.

### 2.4. Data Analysis–Quantitative Content Analysis

As a second step, all transcripts were analyzed using quantitative content analysis. Quantitative content analysis is a research method where target features of a text are systematically categorized and recorded to provide insight into phenomena that involve verbal and/or written communication. This systematic observation and quantification of text patterns are typically referred to as coding [59]. Data obtained using this specific coding provide numerical descriptors of the analyzed text.

In our study, a manual quantitative content analysis by two trained analysts constituted the first phase. We categorized and analyzed 1262 text excerpts. According to the four dimensions identified by Mehl MR [53], the analysis that we performed was instrumental, semantic, broad, and content-focused.

To be more specific, our aim was instrumental. The instrumental analysis focuses mainly on latent content [53]; a text is analyzed for occurrences of a set of themes, the surface form of which is the number of words used to refer to such themes. Therefore, the analysis of word counts yields inferences about the predominance of themes in the texts [60]. The approach was semantic, as the occurrence of the selected themes was not mapped through the bare counting of words and phrases; on the contrary, thanks to the process of interpretation by the human coders, clauses were considered in relation to their conversational meaning. Also, the coding scheme was based on a broad approach since we used an inductive- and phenomenon-oriented coding process. Finally, the focus was on the content instead of on the style.

To standardize the decision-making process and reduce the disagreement among the two coders, a coding book was generated, with a set of instructions about what features to look for in the transcripts [59]. The two coders participated in several training sessions led by the senior researcher to learn to apply the coding scheme.

Themes were selected based upon the literature on the challenges PD patients cope with [61,62] and on their subjective experience [63,64], as well as upon initial reviews of the interview texts. The intention was to focus not only on how participants discussed the motor component of their PD, but also on how they talked about the emotional, cognitive, social, and temporal aspects linked to their diagnosis, history, and, more specifically, experience with the Movement for Parkinson’s program. The Movement for Parkinson’s program content was explored more in-depth by including themes within the dance experience to extract information about specific features of the classes.

Selected themes are reported in Table 2.

## 3. Results

Data were analyzed using LIWC 2015 (Pennebaker Conglomerates; Austin, TX, USA) and STATA 17 (StataCorp, College Station, TX, USA).

Some data focused only on the data from the LIWC software (namely, the summary language variables and linguistic dimensions), while others were specific to the content analysis (i.e., the focus on the references to the Movement for Parkinson’s program). The remaining data were comparable and will be presented together.

### 3.1. Summary Language Variables and Linguistic Dimensions

**Figure 1:**
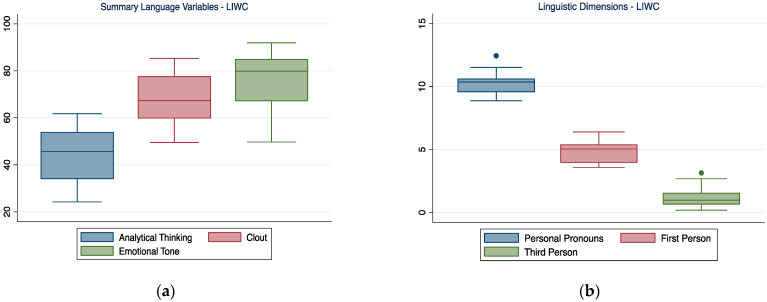
Summary language variables (**a**) and linguistic dimensions (**b**)—LIWC. In the boxplot, the central horizontal line indicates the median, the other two lines indicate the first (lower line) and third (higher line) quartiles, the whiskers indicate the minimum and maximum values, and the dots indicate outliers.

As can be seen in Figure 1, participants tended to produce an emotionally rich narration, as can be derived by the higher score for emotional tone compared to analytic thinking. Clout also seemed to be quite present in participants’ narrations.

When focusing specifically on the use of the pronouns, it was possible to notice how participants kept the narration very personal (high number of personal pronouns) and made more frequent use of 1st person pronouns than 3rd person pronouns.

### 3.2. Emotions

**Figure 2:**
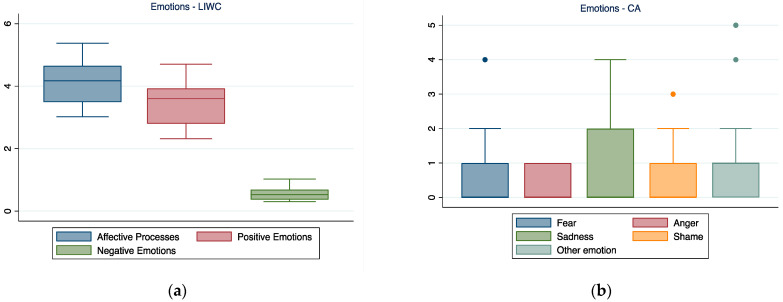
Emotions expressed in the interviews as reflected by LIWC (**a**) and CA (**b**). In the boxplot, the central horizontal line indicates the median, the other two lines indicate the first (lower line) and third (higher line) quartiles, the whiskers indicate the minimum and maximum values, and the dots indicate outliers.

LIWC results highlighted how affective processes were used frequently within the narration, with a predominance of positive emotions emerging from the participants’ talk (see Figure 2).

CA focused mainly on the negative emotions expressed by participants and highlighted how sadness seemed to be the predominant negative emotion that emerged from the interviews.

### 3.3. Social and Cognitive Processes

The LIWC software results highlighted how participants referred almost equally to social and cognitive processes when narrating their experience with PD.

**Figure 3:**
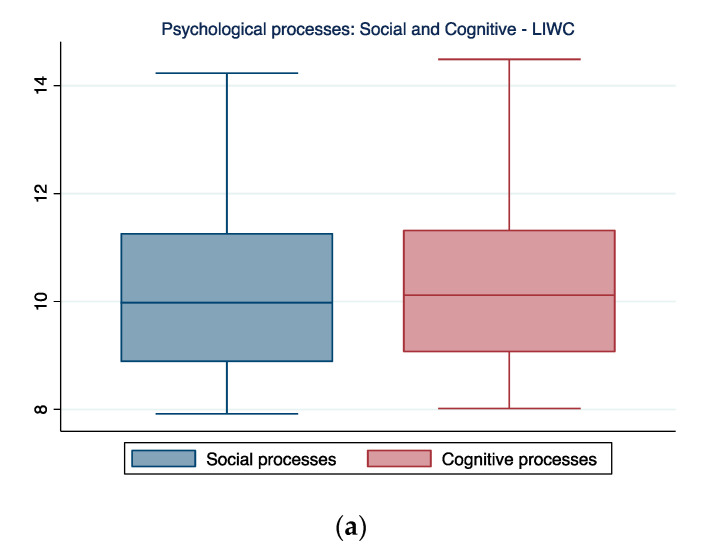

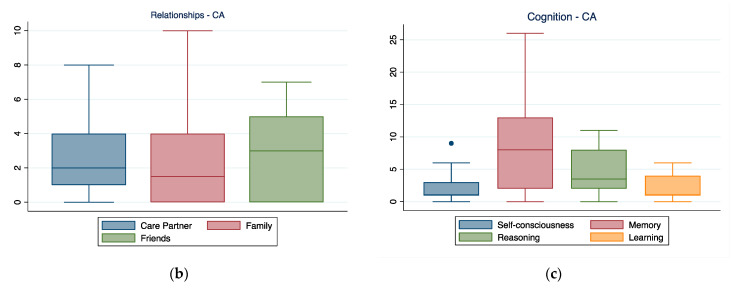
Social and cognitive processes ((**a**) Psychological processes; (**b**) relationships; (**c**) cognition) reflected by the participants’ narrations. In the boxplot, the central horizontal line indicates the median, the other two lines indicate the first (lower line) and third (higher line) quartiles, the whiskers indicate the minimum and maximum values, and the dots indicate outliers.

**Figure 4:**
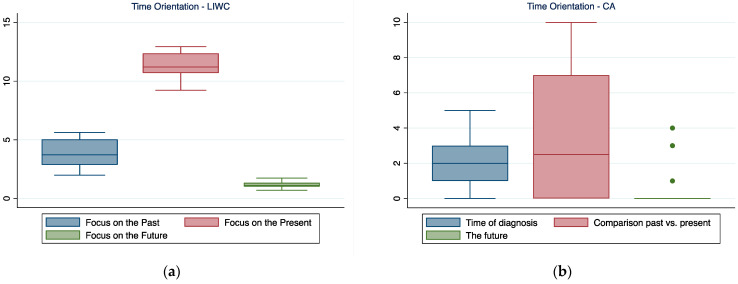
Time orientation emerged by LIWC (**a**) and CA (**b**). In the boxplot, the central horizontal line indicates the median, the other two lines indicate the first (lower line) and third (higher line) quartiles, the whiskers indicate the minimum and maximum values, and the dots indicate outliers.

CA allowed for a more in-depth analysis of each of these categories. As we can see in Figure 3, the patients felt the support of friends and referred to them often, highlighting an appreciation of the social support possibly enhanced through Movement for Parkinson’s. They also used memory processes a lot when cognition was involved, which can be read as a focus on comparing what they are experiencing now with what they have experienced in the past, a feature also confirmed by the data related to “time” reported below (Figure 4).

### 3.4. Time Orientation

When narrating their experience with Parkinson’s, participants focused mainly on the present (LIWC data)—this focus seemed to be aimed at comparing the present situation with how the individuals were before the diagnosis (CA). When focusing on the past, the patients tended to focus mainly on the moment of the diagnosis.

### 3.5. Movement for Parkinson’s

**Figure 5:**
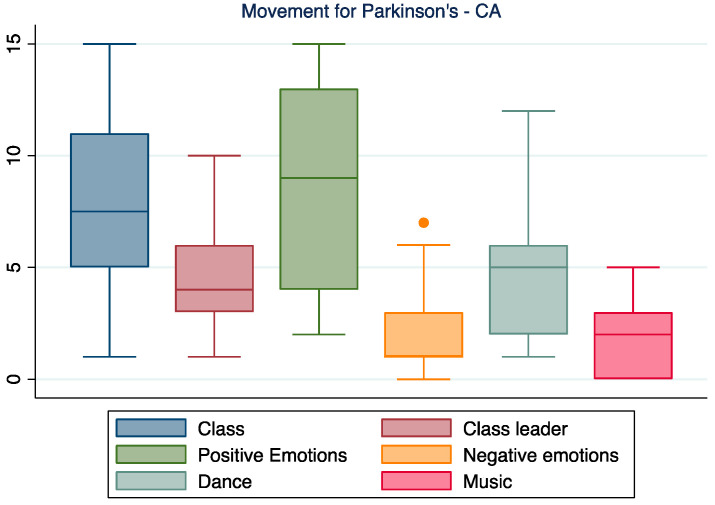
The focus on Movement for Parkinson’s. In the boxplot, the central horizontal line indicates the median, the other two lines indicate the first (lower line) and third (higher line) quartiles, the whiskers indicate the minimum and maximum values, and the dots indicate outliers.

When discussing specifically their experience with the program Movement for Parkinson’s, participants seem to mainly focus on positive emotions linked to the class. The three main components of the class (dance, music, and class leader) seem to have an equal weight in the participants’ positive experience (Figure 5).

## 4. Discussion

The first aim of this study was to explore in-depth the beliefs, subjective experiences, and emotional and cognitive responses of a group of Parkinson’s patients who have attended a dance and music-based class for at least 6 months using an innovative qualitative approach, which combined software-based and human-based text analysis.

We were interested in the effects that the program might have had from a cognitive, emotional, and socio-relational standpoint since these are aspects that have been less explored when dance-based interventions for PD patients have been studied.

Data from our interviews seem to confirm that attending the program had a positive effect on participants’ emotional tone, as reflected by the high score in the emotional tone category. Higher scores in the category of emotional tone have been reported to be linked to a more positive effect [65], a characteristic that was also confirmed by the prevalence of positive emotions when examining the specific affective language used during the interviews. As discussed in the literature, given the need for paying more scientific attention to patients’ everyday emotions in natural contexts, this data is relevant [66]. We know from the literature [67,68] that patients with PD frequently report or exhibit signs of anxiety, apathy, and depressed mood. The fact that our specific sample of patients was able to focus more on positive emotions seems like a strong, if an indirect, sign that a dance- and music-based intervention, such as the one studied here, could have a positive effect on these specific symptoms.

The CA allowed us to focus more specifically on the expression of negative emotions. The first main result highlights how patients were able to show quite a wide range of negative emotions (especially considering that they were less present than the positive ones, as just discussed). Since the literature reports a reduced sensitivity for negative emotions in PD patients [69], especially when a vocal channel is involved (we were analyzing a spoken narration) [10], this information seems to be another marker of a possible beneficial effect of the program—in this case, to improve the expression of a range of negative emotions.

Our data also indicate a prevalence of sadness among the expressed negative emotions, which is not surprising given the fact that a significant proportion of PD patients receive a comorbid diagnoses of depression-apathy or depression-anxiety [70,71].

We were also interested in exploring any possible social-related effects of the program as perceived by our participants. A first interesting result derives from the use of personal pronouns within the narration. Our participants tended to use personal pronouns often, while third-person personal pronouns were used the least. The literature suggests that this pattern is linked to closer in-group social relationships [72]. This general information is confirmed and clarified by the CA, showing how participants talked a great deal about friends. Given that a PD diagnosis is often followed by a loss of social functioning and a significant decrease in the social network of friends, and that traditional interventions for PD have proven to be ineffective [73], our data seem to point towards promising positive outcomes related to dance- and music-based interventions.

Social and cognitive speech seemed to be used at almost the same level in our sample. The literature supports the interesting finding that when individuals are more involved in perspective-taking they also tend to use more “complex” language, reflected by language linked to cognitive processes [72].

Focusing more specifically on cognitive language, the fine-grained CA revealed that memory emerged most prominently when cognitive processes were involved. This appears to be closely related to our data regarding time orientation during the interviews. Since patients focused mainly on the present, it seems the present was used as a comparison between “now” and “before” (the diagnosis), hence, the use of memory-related strategies. Even if memories of the life before the diagnosis are accessed, we found that participants’ focus stays mainly in the present in conjunction with (as discussed above) a predominance of positive affect within the interviews; this seems to imply a mindful attitude of our participants. Since dance and music interventions have been suggested to promote mindfulness [74], we could read this finding as preliminary insight into one of the reasons why Movement for Parkinson’s is so beneficial for participants’ emotional well-being.

This result is linked to our last specific focus, which sought to investigate which aspects of the Movement for Parkinson’s program were the most relevant for our participants. Encouragingly, the results from CA show that when discussing the program specifically, participants focused on the same variables that we were expecting to be affected by participation in the class: positive emotions and the class as a social entity. This confirms that participants do possess a good awareness of the benefits of the program.

Finally, we aimed to assess the type of information derived by a combined approach applied to the interview transcripts, which have been analyzed both by software (LIWC) and by human judges/raters in coding-based CA. We expected to be able to combine these data to dive deeper into the interview content. As made clear in the discussion of our results, this was indeed the case, and this mixed approach to qualitative analysis seems to be a promising strategy for rich interviews.

## 5. Conclusions

This study has some limitations that should be addressed by future studies; we only focused on one program for PD, and we did not follow the participants longitudinally to see if the benefits would change with time of attendance. Moreover, mixed-methods studies should be conducted to collect more detailed information about the different psychological characteristics of the participants. These studies should also include in the design the role of the specific clinical measures linked to PD diagnosis (e.g., time from diagnosis, age at onset, and disease severity).

Our study also did not include a control group, so all of the effects reported here, as derived from the program, are indirect and based on a comparison with the previous literature. For this reason, future studies should seek to compare the effects of a music and dance program to the effects of other therapeutic programs for PD, for example, mindfulness-based programs. Yet, our study adds relevant knowledge to the literature as our main focus was on the individual experience of PD patients. Notably, control groups are not often used in qualitative research [75,76].

It is also important to highlight that this study represents one of the first in-depth attempts at using an innovative qualitative approach aimed at investigating the beliefs, subjective experiences, and emotional and cognitive responses of Parkinson’s patients in an ecological context. The results are promising and support the beneficial effects of programs like Movement for Parkinson’s to promote emotional and social functioning in PD patients.

## Figures and Tables

**Table 1:** LIWC categories used in this study. Adapted from Pennebaker JW, Boyd RL, Jordan K., and Blackburn K. [49].

Category	Examples
*Summary Language Variables*	
Analytical Thinking	/
Clout	/
Emotional tone	/
*Linguistic dimensions*	
Personal pronouns	
1st person	I, me, mine, we, us
3rd person	she, her, him, they, them
*Psychological processes*	
Affective processes	Happy, cried
Positive emotion	Love, nice, sweet
Negative emotion	Hurt, ugly, nasty
Social processes	Mate, talk, daughter, buddy
Cognitive processes	Cause, know, ought
*Time orientation*	
Past focus	Ago, did, talked
Present Focus	Today, is, now
Future Focus	May, will, soon

**Table 2:** Themes used for Content Analysis.

Theme	Subcategories
Relationships	Relationship with the principal care partner Relationship with family Relationship with friends
Cognition	Self-consciousness Memory Reasoning Learning
Emotions	Fear Anger/rage Sadness/sorrow Shame/embarrassment Other emotions
Time	Time of diagnosis Comparison between the present (after being diagnosed with PD) and the past (before being diagnosed) The future
Movement for Parkinson’s	The other participants The group leader Dance Music Positive emotions Negative emotions

## Data Availability

Anonymized data can be obtained by writing to the corresponding author.

## References

[1] Dorsey E.R., Holloway R.G., Ravina B.M. (2006). Biomarkers in Parkinson’s disease. Expert Rev. Neurother..

[2] Rossi A., Berger K., Chen H., Leslie D., Mailman R.B., Huang X. (2018). Projection of the prevalence of Parkinson’s disease in the coming decades: Revisited. Mov. Disord..

[3] Marras C., Beck J., Bower J., Roberts E., Ritz B., Ross G., Abbott R., Savica R., Van Den Eeden S., Willis A. (2018). Prevalence of Parkinson’s disease across North America. Npj Parkinson’s Dis..

[4] Foltynie T., Langston J.W. (2018). Therapies to slow, stop, or reverse Parkinson’s disease. J. Parkinson’s Dis..

[5] Witt K., Kalbe E., Erasmi R., Ebersbach G. (2017). Nonpharmacological treatment procedures for Parkinson’s disease. Der. Nervenarzt..

[6] Šumec R., Filip P., Sheardová K., Bareš M. (2015). Psychological benefits of nonpharmacological methods aimed for improving balance in Parkinson’s disease: A systematic review. Behav. Neurol..

[7] Fancourt D., Finn S. (2019). What Is the Evidence on the Role of the Arts in Improving Health and Well-Being? A Scoping Review.

[8] Koch S., Kunz T., Lykou S., Cruz R. (2014). Effects of dance movement therapy and dance on health-related psychological outcomes: A meta-analysis. Arts Psychother..

[9] Bearss K.A., DeSouza J.F. (2021). Parkinson’s Disease Motor Symptom Progression Slowed with Multisensory Dance Learning over 3-Years: A Preliminary Longitudinal Investigation. Brain Sci..

[10] Biassoni F., Gnerre M., Malaspina E., Di Tella S., Anzuino I., Baglio F., Silveri M.C. (2022). How does prosodic deficit impact naïve listeners recognition of emotion? An analysis with speakers affected by Parkinson’s disease. Psychol. Lang. Commun..

[11] Park A., Stacy M. (2009). Non-motor symptoms in Parkinson’s disease. J. Neurol..

[12] Zhang Q., Hu J., Wei L., Jia Y., Jin Y. (2019). Effects of dance therapy on cognitive and mood symptoms in people with Parkinson’s disease: A systematic review and meta-analysis. Complement. Ther. Clin. Pract..

[13] Alpert P.T. (2011). The health benefits of dance. Home Health Care Manag. Pract..

[14] Westbrook B.K., McKibben H. (1989). Dance/movement therapy with groups of outpatients with Parkinson’s disease. Am. J. Danc. Ther..

[15] Earhart G.M. (2009). Dance as therapy for individuals with Parkinson disease. Eur. J. Phys. Rehabil. Med..

[16] McNeely M., Duncan R., Earhart G. (2015). A comparison of dance interventions in people with Parkinson disease and older adults. Maturitas.

[17] Pereira A.P.S., Marinho V., Gupta D., Magalhães F., Ayres C., Teixeira S. (2019). Music therapy and dance as gait rehabilitation in patients with Parkinson disease: A review of evidence. J. Geriatr. Psychiatry Neurol..

[18] Hackney M.E., Kantorovich S., Levin R., Earhart G.M. (2007). Effects of tango on functional mobility in Parkinson’s disease: A preliminary study. J. Neurol. Phys. Ther..

[19] Hackney M.E., Earhart G.M. (2009). Backward walking in Parkinson’s disease. Mov. Disord. Off. J. Mov. Disord. Soc..

[20] Li F., Harmer P., Fitzgerald K., Eckstrom E., Stock R., Galver J., Maddalozzo G., Batya S.S. (2012). Tai chi and postural stability in patients with Parkinson’s disease. N. Engl. J. Med..

[21] Aguiar L.P.C., da Rocha P.A., Morris M. (2016). Therapeutic dancing for Parkinson’s disease. Int. J. Gerontol..

[22] Kiepe M.-S., Stöckigt B., Keil T. (2012). Effects of dance therapy and ballroom dances on physical and mental illnesses: A systematic review. Arts Psychother..

[23] Batson G. (2010). Feasibility of an intensive trial of modern dance for adults with Parkinson disease. Complement. Health Pract. Rev..

[24] Heiberger L., Maurer C., Amtage F., Mendez-Balbuena I., Schulte-Mönting J., Hepp-Reymond M.-C., Kristeva R. (2011). Impact of a weekly dance class on the functional mobility and on the quality of life of individuals with Parkinson’s disease. Front. Aging Neurosci..

[25] Eyigor S., Karapolat H., Durmaz B., Ibisoglu U., Cakir S. (2009). A randomized controlled trial of Turkish folklore dance on the physical performance, balance, depression and quality of life in older women. Arch. Gerontol. Geriatr..

[26] Duncan R.P., Earhart G.M. (2014). Are the effects of community-based dance on Parkinson disease severity, balance, and functional mobility reduced with time? A 2-year prospective pilot study. J. Altern. Complement. Med..

[27] Lee N.-Y., Lee D.-K., Song H.-S. (2015). Effect of virtual reality dance exercise on the balance, activities of daily living, and depressive disorder status of Parkinson’s disease patients. J. Phys. Ther. Sci..

[28] McGill A., Houston S., Lee R.Y. (2014). Dance for Parkinson’s: A new framework for research on its physical, mental, emotional, and social benefits. Complement. Ther. Med..

[29] Shanahan J., Morris M.E., Bhriain O.N., Volpe D., Lynch T., Clifford A.M. (2017). Dancing for Parkinson disease: A randomized trial of Irish set dancing compared with usual care. Arch. Phys. Med. Rehabil..

[30] McKee K.E., Hackney M.E. (2013). The effects of adapted tango on spatial cognition and disease severity in Parkinson’s disease. J. Mot. Behav..

[31] Warburton E.C., Wilson M., Lynch M., Cuykendall S. (2013). The cognitive benefits of movement reduction: Evidence from dance marking. Psychol. Sci..

[32] Kalyani H., Sullivan K., Moyle G., Brauer S., Jeffrey E.R., Kerr G. (2019). Impacts of dance on cognition, psychological symptoms and quality of life in Parkinson’s disease. NeuroRehabilitation.

[33] McGarry L.M., Russo F.A. (2011). Mirroring in dance/movement therapy: Potential mechanisms behind empathy enhancement. Arts Psychother..

[34] Behrends A., Müller S., Dziobek I. (2012). Moving in and out of synchrony: A concept for a new intervention fostering empathy through interactional movement and dance. Arts Psychother..

[35] Kattenstroth J.-C., Kolankowska I., Kalisch T., Dinse H.R. (2010). Superior sensory, motor, and cognitive performance in elderly individuals with multi-year dancing activities. Front. Aging Neurosci..

[36] Hobeika L., Samson S. (2020). Why do music-based interventions benefit persons with neurodegenerative disease?. Music and the Aging Brain.

[37] Zhang M.Y. (2022). Dance for AD: A Multicomponent Intervention to Mitigate the Negative Effects of Alzheimer’s Disease.

[38] Lazarou I., Parastatidis T., Tsolaki A., Gkioka M., Karakostas A., Douka S., Tsolaki M. (2017). International ballroom dancing against neurodegeneration: A randomized controlled trial in Greek community-dwelling elders with mild cognitive impairment. Am. J. Alzheimers Dis. Dement..

[39] Barnish M.S., Barran S.M. (2020). A systematic review of active group-based dance, singing, music therapy and theatrical interventions for quality of life, functional communication, speech, motor function and cognitive status in people with Parkinson’s disease. BMC Neurol..

[40] Rocha P.A., Slade S.C., McClelland J., Morris M.E. (2017). Dance is more than therapy: Qualitative analysis on therapeutic dancing classes for Parkinson’s. Complement. Ther. Med..

[41] Diaz Abrahan V., Justel N., Shifres F. (2022). Musical improvisation: A mixed methods study on social interactions in younger and older adults. Nord. J. Music. Ther..

[42] Boster J.B., Spitzley A.M., Castle T.W., Jewell A.R., Corso C.L., McCarthy J.W. (2021). Music improves social and participation outcomes for individuals with communication disorders: A systematic review. J. Music Ther..

[43] Prewitt C.M., Charpentier J.C., Brosky J.A., Urbscheit N.L. (2017). Effects of dance classes on cognition, depression, and self-efficacy in Parkinson’s disease. Am. J. Danc. Ther..

[44] Iannello P., Biassoni F., Bertola L., Antonietti A., Caserta V.A., Panella L. (2018). The role of autobiographical story-telling during rehabilitation among hip-fracture geriatric patients. Eur. J. Psychol..

[45] Zimmermann M. (2012). Narrating stroke: The life-writing and fiction of brain damage. Med. Humanit..

[46] Jackson J.C., Watts J., List J.-M., Puryear C., Drabble R., Lindquist K.A. (2021). From text to thought: How analyzing language can advance psychological science. Perspect. Psychol. Sci..

[47] Tausczik Y.R., Pennebaker J.W. (2010). The psychological meaning of words: LIWC and computerized text analysis methods. J. Lang. Soc. Psychol..

[48] Renz S.M., Carrington J.M., Badger T.A. (2018). Two strategies for qualitative content analysis: An intramethod approach to triangulation. Qual. Health Res..

[49] Pennebaker J.W., Boyd R.L., Jordan K., Blackburn K. (2015). The Development and Psychometric Properties of LIWC2015. https://repositories.lib.utexas.edu/bitstream/handle/2152/31333/LIWC2015_LanguageManual.pdf.

[50] Mergenthaler E., Bucci W. (1999). Linking verbal and non-verbal representations: Computer analysis of referential activity. Br. J. Med. Psychol..

[51] Viney L.L. (1983). The assessment of psychological states through content analysis of verbal communications. Psychol. Bull..

[52] Weintraub W. (1989). Verbal Behavior in Everyday Life.

[53] Mehl M.R., Eid M., Diener E. (2006). Quantitative Text Analysis. Handbook of Multimethod Measurement in Psychology.

[54] Malterud K., Siersma V.D., Guassora A.D. (2016). Sample size in qualitative interview studies: Guided by information power. Qual. Health Res..

[55] Morse J. (2020). The changing face of qualitative inquiry. Int. J. Qual. Methods.

[56] Pennebaker J.W. (2011). The secret life of pronouns. New Sci..

[57] Pennebaker J.W., Francis M.E. (1996). Cognitive, emotional, and language processes in disclosure. Cogn. Emot..

[58] Barrett D. (2020). Dreams about COVID-19 versus normative dreams: Trends by gender. Dreaming.

[59] Coe K., Scacco J.M. (2017). Content analysis, quantitative. Int. Encycl. Commun. Res. Methods.

[60] Roberts C.W. (2000). A conceptual framework for quantitative text analysis. Qual. Quant..

[61] Kuopio A.M., Marttila R.J., Helenius H., Toivonen M., Rinne U.K. (2000). The quality of life in Parkinson’s disease. Mov. Disord. Off. J. Mov. Disord. Soc..

[62] Opara J., Brola W., Leonardi M., Błaszczyk B. (2012). Quality of life in Parkinsons Disease. J. Med. Life.

[63] Rutten S., van den Heuvel O.A., de Kruif A., Schoonmade L.J., Schumacher E.I., Vermunt K., Hagen R., van Wegen E.E., Rutten K. (2021). The subjective experience of living with Parkinson’s disease: A meta-ethnography of qualitative literature. J. Parkinson’s Dis..

[64] Hanff A.-M., Leist A.K., Fritz J.V., Pauly C., Krüger R., Halek M., Consortium N.-P. (2022). Determinants of Self-Stigma in People with Parkinson’s Disease: A Mixed Methods Scoping Review. J. Parkinson’s Dis..

[65] Cohn M.A., Mehl M.R., Pennebaker J.W. (2004). Linguistic markers of psychological change surrounding September 11, 2001. Psychol. Sci..

[66] Sotgiu I., Rusconi M.L. (2013). Investigating emotions in Parkinson’s disease: What we know and what we still don’t know. Front. Psychol..

[67] Nègre-Pagès L., Grandjean H., Lapeyre-Mestre M., Montastruc J.L., Fourrier A., Lépine J.P., Rascol O., Group D.S. (2010). Anxious and depressive symptoms in Parkinson’s disease: The French cross-sectionnal DoPaMiP study. Mov. Disord..

[68] Siri C., Cilia R., De Gaspari D., Villa F., Goldwurm S., Marco C., Pezzoli G., Antonini A. (2010). Psychiatric symptoms in Parkinson’s disease assessed with the SCL-90R self-reported questionnaire. Neurol. Sci..

[69] Dara C., Monetta L., Pell M.D. (2008). Vocal emotion processing in Parkinson’s disease: Reduced sensitivity to negative emotions. Brain Res..

[70] Lubomski M., Davis R.L., Sue C.M. (2020). Depression in Parkinson’s disease: Perspectives from an Australian cohort. J. Affect. Disord..

[71] Oguru M., Tachibana H., Toda K., Okuda B., Oka N. (2010). Apathy and depression in Parkinson disease. J. Geriatr. Psychiatry Neurol..

[72] Van Swol L.M., Ahn P.H., Prahl A., Gong Z. (2021). Language Use in Group Discourse and Its Relationship to Group Processes. SAGE Open.

[73] Perepezko K., Hinkle J.T., Shepard M.D., Fischer N., Broen M.P., Leentjens A.F., Gallo J.J., Pontone G.M. (2019). Social role functioning in Parkinson’s disease: A mixed-methods systematic review. Int. J. Geriatr. Psychiatry.

[74] Sheppard A., Broughton M.C. (2020). Promoting wellbeing and health through active participation in music and dance: A systematic review. Int. J. Qual. Stud. Health Well-Being.

[75] Busetto L., Wick W., Gumbinger C. (2020). How to use and assess qualitative research methods. Neurol. Res. Pract..

[76] Flick U. (2004). Design and process in qualitative research. Companion Qual. Res..

